# Supplementary material for: Chromothripsis during telomere crisis is independent of NHEJ, and consistent with a replicative origin
Source: Genome Res. 2019 May;29(5):737–49. doi: 10.1101/gr.240705.118 (PMC6499312; doi:10.1101/gr.240705.118)
Supplement: Supplemental Material [file supp_gr.240705.118_Supplemental_file_1.zip › contigs/annotated_contigs/DB45/contig.2.DB45_length_764_mean_cov_10.5366492147.docx]

**DB45_length_764_mean_cov_10.5366492147**

```
TCCAGTTCTAAAGGTCCTGATACATTACCTGGAGTCAACTGTCTCATTTAAACTGTCTTCAGTACAGCTCGGCACAGTTCATCAGTTAA
 >chr2:134790766-134791195 - E=3e-246 p=1e-02
TCAGGCCCTGGGTTGAACAGTGGGTAGGACCCAAGGCTGTGCTCTCAGGAGGTGGGGAAAGCTATTGCACCTCTTTTTTGAACGCGGAT

TTGAATTTTGGAAGATACAAGTAAGTTTCTAATTTGGAGAGTAGGGTGGTATGTACCTTGCAGAGAAAACCTTCTAGGTAGAAGGATTT

TAAAATGTGTCCCAGAAGATTGGTGAATCAGGTCCAAAATCCTGGGTTGGCAGCAGTTTCATATGTTATTAAATCTTGACTTTAGAGTT

GAAAGGAATTTCCCACTGGAAAAAACATAACAAAAGCTGAAAACTTGGCACTTGACTAGAAAATCAAGAAGAG|AT|GCCCCTGATGCC
                                                                            >chr2:13478
ATTCCACTGAGGCCGAGGGTCCCAGCTGCTTCCCCACTCTGGACCCTTGAAGCAGCACAGGATGCTCCATGGACCGCTGGTCAGCTGTC
9259-134789592 - E=2e-188
TGTTTCTAACTTGGAATTGTTTGCCTCTACTAAATCAGCCACTCTGTGCGCAATCTGGACAAGTTGTTTACCTTCTGAGCTTTCAGGTT

TTTTTGCCTGCAAAATGAGGGAGTTAGTTGGTCAGACCCAGTGGATGGCAGCTCTGGCTGTGGGTGAAACCACAGGTGAAACTCACCTG

GGAGGATTTAACCTGAATAGGGAAGTAGGTGCTGTAGCAAACATTTTTCAGGGC
```
